# Evaluation of *DNMT3A* genetic polymorphisms as outcome predictors in AML patients

**DOI:** 10.18632/oncotarget.11143

**Published:** 2016-08-09

**Authors:** Xiao-Qing Yuan, Dao-Yu Zhang, Han Yan, Yong-Long Yang, Ke-Wei Zhu, Yan-Hong Chen, Xi Li, Ji-Ye Yin, Xiao-Lin Li, Hui Zeng, Xiao-Ping Chen

**Affiliations:** ^1^ Department of Clinical Pharmacology, Xiangya Hospital, Central South University, Changsha 410008, P. R. China; ^2^ Institute of Clinical Pharmacology, Central South University, Hunan Key Laboratory of Pharmacogenetics, Changsha 410078, P. R. China; ^3^ Department of Pharmacy, Haikou People's Hospital and Affiliated Haikou Hospital of Xiangya Medical School, Central South University, Haikou 570311, P. R. China; ^4^ Department of Hematology, Xiangya Hospital, Central South University, Changsha 410008, P. R. China; ^5^ Hunan Province Cooperation Innovation Center for Molecular Target New Drug Study, Hengyang 421001, P. R. China

**Keywords:** DNMT3A, polymorphism, AML, chemosensitivity, prognosis

## Abstract

*DNMT3A* mutation is known as a recurrent event in acute myelogenous leukemia (AML) patients. However, association between *DNMT3A* genetic polymorphisms and AML patients' outcomes is unknown. *DNMT3A* 11 SNPs (rs11695471, rs2289195, rs734693, rs2276598, rs1465825, rs7590760, rs13401241, rs7581217, rs749131, rs41284843 and rs7560488) were genotyped in 344 diagnostic non-FAB-M3 AML patients from southern China. Patients underwent combined chemotherapy with cytarabine and anthracyclines. *DNMT3A* mRNA expression was analyzed in PBMCs from randomly selected AML patients. Multivariate analysis and combined genotype analysis showed that rs2276598 was associated with increased while rs11695471 and rs734693 were associated with decreased chemosensitivity (*P*<0.05), while rs11695471 (worse for OS), rs2289195 (favorable for OS and DFS) and rs2276598 (favorable for DFS) were significantly associated with disease prognosis (*P*<0.05). In conclusion, *DNMT3A* polymorphisms may be potential predictive markers for AML patients' outcomes, which might improve prognostic stratification of AML.

## INTRODUCTION

Human acute myelogenous leukemia (AML) mainly occurring in adults is a genetically and epigenetically heterogeneous disorder, which presents a high morbidity and mortality all over the world including in China [1–5]. Despite genetic heterogeneity of the disease, patients excluding FAB-M3 AML have been treated with similar combinations of cytarabine and anthracyclines for over 4 decades with little improvement in clinical outcome [3, 4, 6, 7].

*DNA (cytosine-5-)-methyltransferase 3 alpha* (*DNMT3A*) gene encodes a de nove methyltransferase, which was known as one of the most frequently mutated genes in AML [8–12]. *DNMT3A* mutation, probably a driver mutation, shows high stability from AML initiation up to relapse [8, 13–17]. *DNMT3A* R882 mutation, the main form of *DNMT3A* mutations [10, 18–21], acts commonly as an unfavorable prognostic marker in AML patients [11, 21–27].

Previous literatures elicited that *DNMT3A* polymorphisms were mainly associated with susceptibility of solid tumors, such as gastric carcinoma [28–31], colorectal cancer [32], hepatocellular carcinoma [33], ovarian cancer [34, 35] and breast cancer [36, 37] Besides, *DNMT3A* polymorphisms can lead to changes in methylation of downstream genes, such as *LINE-1* and the imprinted gene *PEG3* [38–40]. So far, there is no report regarding association between *DNMT3A* polymorphisms and the outcome of AML patients, although polymorphisms in some genes are shown to play vital roles in initiation and development of hematologic malignancies [41–44].

In consideration that prognostic stratification was critical for determination of therapeutic regimens and improving survival for AML patients [8, 45–48], we determined the associations of *DNMT3A* single nucleotide polymorphisms (SNPs) with the prognosis of AML patients from southern China. And the associations of *DNMT3A* SNPs with the patients' chemosensitivity were also assessed.

## RESULTS

### The baseline characteristics of patients

The entire study population consisted of 352 diagnostic non-FAB-M3 AML patients who received induction chemotherapy with cytarabine and anthracyclines and were evaluable for response. Genotypes were unavailable from 8 patients for all eleven SNPs. The rest of 344 subjects were eligible for this study. Baseline characteristics in overall subjects were shown in Table 1. Undergoing a median follow-up of 442 days (range, 15-1500 days), 60% patients achieved a complete remission (CR). All the subjects were self-reported Chinese ancestry. The median age was 42 years (range, 14-79 years), and 45% were women. Forty-four patients (12.8%) harbored *FLT3*-ITD mutation, 42 patients (12.2%) harbored *NPM1* mutation, and 27 patients (7.8%) harbored *DNMT3A* R882 mutations (23 R882H mutation and 4 R882C mutation). Interestingly, patients positive for *NPM1* mutations were usually accompanied by positive mutations for *FLT3*-ITD and *DNMT3A* R882 mutations (Supplementary Table S2). Thirty-nine patients (11.3%) received hematopoietic cell transplantation (HCT). For patients received HCT, survival data was censored at the time of undergoing transplantation.

**Table 1:** The baseline characteristics in entire AML patients

Characteristics	Available patients (n,%)	Median (Range)	Missing (n,%)
**Sex**			
**Male**	188(54.7)	—	
**Female**	156(45.3)	—	
**Age, years**	344(100.0)	42(14-79)	
**BSA**	302(87.8)	1.6(1.2-2.1)	42(12.2)
**BMI**	302(87.8)	22.4(15.6-35.1)	42(12.2)
**FAB classification**			
**M0**	1(0.3)	—	
**M1**	20(5.8)	—	
**M2**	165(48.0)	—	
**M4**	50(14.5)	—	
**M5**	63(18.3)	—	
**M6**	5(1.5)	—	
**M7**	1(0.3)	—	
**Other AML**	37(10.8)	—	
**Mix(AML+ALL)**	2(0.6)	—	
***FLT3*-ITD mutation**			2(0.6)
**Positive**	44(12.8)	—	
**Negative**	298(86.6)	—	
***NPM1* mutation**			2(0.6)
**Positive**	42(12.2)	—	
**Negative**	300(87.2)	—	
**Risk stratifications^a^**			
**Low risk**	62(18.0)	—	
**Intermediate risk**	168(48.8)	—	
**High risk**	114(33.2)	—	
***DNMT3A* R882 mutation**			
**Positive**	27(7.8)	—	
**Negative**	317(92.2)	—	
**Chemotherapy regimens**			
**Mitoxantrone + AraC**	88(25.6)	—	
**AraC + ACLA + G-CSF**	107(31.1)	—	
**Daunorubicin + AraC**	42(12.2)	—	
**Idarubicin + AraC**	47(13.7)	—	
**THP + AraC**	29(8.4)	—	
**Others**	31(9.0)	—	
**BM blasts, %**	329(95.6)	70.0(2.0-99.0)	15(4.4)
**PB blasts, %**	277(80.5)	60.0(6.0-97.0)	67(19.5)
**WBC, ×10^9^/L**	344(100.0)	14.5(0.5-436.2)	
**RBC, ×10^12^/L**	343(99.8)	2.2(0.6-4.9)	1(0.2)
**Hemoglobin, g/L**	344(100.0)	71.0(27.0-155.0)	
**Platelets, ×10^9^/L**	344(100.0)	30.0(3.0-546.0)	
**Neutrophils, ×10^9^/L**	342(99.4)	2.5(0.0-250.0)	2(0.6)
**LDH, U/L**	336(97.7)	355.6(44.7-7286.0)	8(2.3)
**Treatment response**			
**Early death**	33(9.6)	—	
**CR after 1st course**	107(31.1)	—	
**CR after 2nd course**	98(28.5)	—	
**Partial remission**	37(10.8)	—	
**No remission**	69(20.1)	—	
**CR after 2 courses**			
**Yes**	205(59.6)		
**No**	139(40.4)		
**HCT**			
**Yes**	39(11.3)	—	
**No**	305(88.7)	—	

a The risk stratification based on validated cytogenetics and molecular data was classified according to NCCN Guidelines Version 1.2015 Acute Myeloid Leukemia. **Abbreviations:** BSA, body surface area; BMI, Body Mass Index; FAB, French-American-British (classification); R882, Arginine 882; FLT3-ITD, fms-like tyrosine kinase3-intenal tandem duplication; BM, bone marrow; PB, peripheral blood; WBC, white blood cell; RBC, red blood cell; LDH, lactate dehydrogenase; AraC, cytarabine; ACLA, aclarubicin; G-CSF, granulocyte-colony stimulating factor; THP, pirarubicin; CR, complete remission; HCT, hematopoietic cell transplantation.

### Genotype Information and linkage disequilibrium analysis

**Figure 1:**
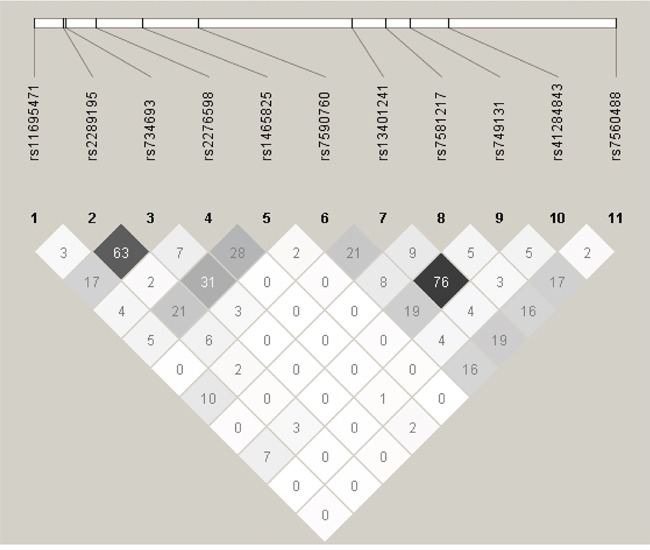
Pairwise linkage disequilibrium of the 11 *DNMT3A* SNPs in the studied AML patients The linkage disequilibrium (LD) of *DNMT3A* polymorphisms at 11 loci were analyzed using Haploview software and indicated by r^2^ value (%).

Eleven SNPs (rs11695471, rs2289195, rs734693, rs2276598, rs1465825, rs7590760, rs13401241, rs7581217, rs749131, rs41284843 and rs7560488) at *DNMT3A* gene locus were chosen from a Deep Catalog of Human Genetic Variation database--1000 Genomes Project (available at http://www.1000genomes.org/home). Table 2 showed detailed information regarding these 11 SNPs. Rs2276598 (Leu 422 Leu) and rs41284843 (Pro 9 Pro) were synonymous variations, while the other nine SNPs were intron variations. The minor allele frequencies (MAF) of *DNMT3A* polymorphisms in this study were comparable to those of Han population in Southern China (CHS) from 1000 Genomes Project. Hardy–Weinberg equilibrium of the genotype distribution was discovered for 11 SNPs in this study (Table 2). The linkage disequilibrium (LD) of *DNMT3A* polymorphisms at 11 loci were shown in Figure 1. There was no obvious LD between each pair of the 11 SNPs (r^2^ < 0.8).

**Table 2:** Characteristics of 11 selected tag SNPs from *DNMT3A*

SNP	Position	MAF (ref^a^)	Alleles	Variation	*P*^b^
**rs11695471**	2:25234839	0.083(0.10)	T:A	Intron22	1.0000
**rs2289195**	2:25240614	0.268(0.29)	G:A	Intron18	0.5917
**rs734693**	2:25241002	0.345(0.37)	C:T	Intron17	0.3794
**rs2276598**	2:25246633	0.330(0.30)	C:T	Exon10 (Leu422Leu)	0.4515
**rs1465825**	2:25255584	0.373(0.38)	T:C	Intron6	0.6282
**rs7590760**	2:25266314	0.301(0.34)	G:C	Intron6	0.0287
**rs13401241**	2:25295601	0.310(0.24)	C:A	Intron3	1.0000
**rs7581217**	2:25302075	0.423(0.45)	C:T	Intron2	0.3109
**rs749131**	2:25306755	0.359(0.30)	A:C	Intron2	0.3950
**rs41284843**	2:25313958	0.086(0.09)	G:A	Exon2(Pro9Pro)	0.5456
**rs7560488**	2:25345952	0.178(0.12)	T:C	5-Flanking	0.4571

a ref indicates the MAF value of CHS from 1000 Genomes.

b *P* value for Hardy–Weinberg equilibrium analysis. **Abbreviations**: SNP, single nucleotide polymorphism; MAF, minor allele frequency.

### Associations between individual SNPs and chemosensitivity status in AML patients

#### Univariate analysis

Associations of *DNMT3A* SNPs with chemosensitivity after first or second induction chemotherapy were shown in Table 3. Chemosensitivity was defined as good response (GR) or poor response (PR) in this study. Significant associations between three SNPs (rs11695471, rs2276598, rs734693) and good response (GR) ratio were observed. Rs734693 CT/TT genotypes showed a trend of lower GR ratio (*P*=0.093, dominant model) with an OR of 1.50 (95% CI: 0.93-2.41). A higher GR ratio was observed for rs2276598 CT/TT genotypes (*P*=0.046, dominant model) with an OR of 0.62 (95% CI: 0.39-0.99). Rs11695471 AT/AA genotypes also showed a lower GR ratio (*P*=0.041, dominant model) with an OR of 1.84 (95% CI: 1.02-3.33). No significant associations of other 8 SNPs with GR ratio were found (Table 3).

**Table 3:** Univariate and Logistic regression analysis of associations between *DNMT3A* SNPs and chemosensitivity in entire AML patients

SNP	Genotype	N	*P*	OR (95% CI)^a^	*P ^a^*
rs11695471	TA+AA v TT	344	**0.041**	2.012 (1.060-3.819)	**0.033**
rs2289195	GA+AA v GG	343	0.584	0.755 (0.265-2.153)	0.599
rs734693	CT+TT v CC	344	0.093	1.691 (1.004-2.849)	**0.048**
rs2276598	CT+TT v CC	337	**0.046**	0.587 (0.347-0.995)	**0.048**
rs1465825	TC+CC v TT	342	0.654	0.960 (0.570-1.618)	0.878
rs7590760	GC+CC v GG	342	0.553	0.910 (0.410-2.020)	0.816
rs13401241	CA+AA v CC	342	0.927	1.013 (0.606-1.694)	0.959
rs7581217	CT+TT v CC	342	0.534	0.901 (0.527-1.540)	0.702
rs749131	AC+CC v AA	341	0.876	1.035 (0.615-1.741)	0.898
rs41284843	GA+AA v GG	340	0.382	1.362 (0.721-2.574)	0.342
rs7560488	TC+CC v TT	337	0.916	1.017 (0.589-1.758)	0.951

**NOTE:** Bold font indicates statistical significance.

a Adjusted for gender, age and FAB classification. **Abbreviations:** SNP, single nucleotide polymorphism; OR, odds ratio; CI, confidence interval.

The characteristics of AML patients between or among genotypes of rs11695471, rs2276598, rs734693 and other eight SNPs were described (data not shown). These characteristics, such as gender, age or FAB classification at diagnosis showed no significant difference between or among genotypes of these SNPs.

#### Logistic regression analysis

In order to ascertain independent predictive factors for chemosensitivity, except for *DNMT3A* SNP rs11695471, rs2276598 and rs734693, covariates including gender, age and FAB classification were included in a logistic regression model. The rationale for including these variables was that they showed significant association with chemosensitivity in univariate analysis (*P*<0.05, data not shown). Results of logistic regression analysis showed that rs2276598 CT/TT carriers showed a significantly higher GR ratio (OR=0.587; 95% CI: 0.347-0.995; *P*=0.048] as compared to the CC homozygotes, whereas rs734693 CT/TT carriers (OR=1.691; 95% CI: 1.004-2.849; *P*=0.048) and rs11695471 AT/AA carriers (OR=2.012; 95% CI: 1.060-3.819; *P*=0.033) showed markedly lower GR ratio as compared with rs734693 CC genotype and rs11695471 TT genotype, respectively (Table 3).

### A combined genotype analysis of rs11695471, rs2276598 and rs734693 for AML chemosensitivity

*DNMT3A* polymorphisms (rs11695471, rs2276598 and rs734693) were shown to exert individual effect on chemosensitivity in AML, but their combined effect had not been elucidated yet. Hence, a *DNMT3A* combined genotype score model was generated and results of combined genotype analysis were presented in Table 4. Chi-square test indicated that a defined favorable response group (composite score 1, 2 or 3) had a higher GR ratio compared with a defined unfavorable response group (composite score 0) (favorable group: 73.1% *vs* unfavorable group: 47.1%, *P*=0.002). Adjusted for covariates including gender, age and FAB classification, binary logistic regression analysis indicated that the defined favorable response group from a *DNMT3A* combined genotype score model had a higher GR ratio (OR=0.242; 95% CI: 0.109-0.536; *P*=0.001).

**Table 4:** Combined analysis of the association between rs11695471, rs2276598 and rs734693 genotypes and AML chemosensitivity

Composite Score^a^	GR ratio	N	*P*	OR (95% CI)^b^	*P*^b^
Composite score 0	47.1%	34		1.00 (reference)	
composite score 1	70.6%	85	**0.017**	0.315 (0.130-0.763)	**0.011**
composite score 2	73.6%	121	**0.004**	0.248 (0.105-0.587)	**0.001**
composite score 3	76.3%	97	**0.002**	0.244 (0.101-0.588)	**0.002**
composite score1/2/3	73.1%	305	**0.002**	0.242 (0.109-0.536)	**0.001**

**NOTE:** Bold font indicates statistical significance.

**Abbreviations:** SNP, single nucleotide polymorphism; GR, good response; OR, odd ratio; CI, confidence interval.

a A *DNMT3A* combined genotype score model was created by figuring up the genotyped data of SNPs rs11695471, rs734693 and rs2276598. Score 1 indicated favorable alleles (i.e. rs11695471 TT genotype, or rs734693 CC genotype, or rs2276598 CT/TT genotype for chemosensitivity) and score 0 indicated unfavorable alleles (i.e. rs11695471 TA/AA genotype, or rs734693 CT/TT genotype, or rs2276598 CC genotype for chemosensitivity). After adding up these scores, four composite score groups were generated: composite score 0, composite score 1, composite score 2 and composite score 3. Two chemotherapy response groups were also defined: unfavorable response (composite score 0) and favorable response (composite score 1/2/3).

b Adjusted for gender, age and FAB classification.

### *DNMT3A* genetic polymorphisms predicted disease prognosis in AML patients

**Figure 2:**
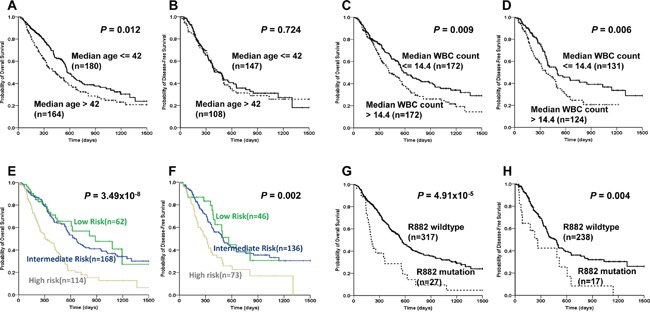
Associations of age, WBC count, risk stratification and *DNMT3A* R882 mutation with disease survivals in AML patients Kaplan-Meier evaluation of OS **A.** and DFS **B.** based on the median age in the entire AML cohort. Kaplan-Meier evaluation of OS **C.** and DFS **D.** based on median WBC count in the entire AML cohort. Kaplan-Meier evaluation of OS **E.** and DFS **F.** based on risk stratification in the entire AML cohort. Kaplan-Meier evaluation of OS **G.** and DFS **H.** based on the status of *DNMT3A* R882 mutation in the entire AML cohort. Patients failed to achieve CR were omitted in the DFS analysis.

Survival analysis verified that the *DNMT3A* R882 mutation AML patients showed markedly shorter median OS and DFS compared with R882 wildtype patients (R882 mutation: 212 days *vs* R882 wildtype: 560 days, *P*<0.0001 for OS; R882 mutation: 228 days *vs* R882 wildtype: 469 days, *P*=0.004 for DFS, Figure 2G, 2H).

### Univariate analysis for disease prognosis

**Figure 3:**
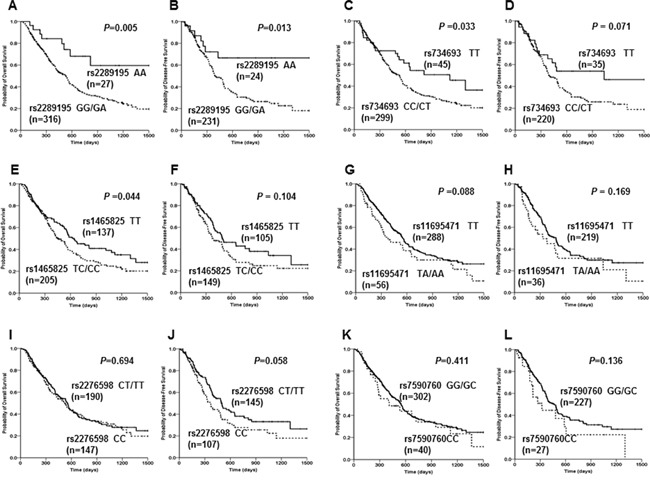
Associations of *DNMT3A* rs2289195, rs7346693, rs1465825, rs11695471, rs2276598 and rs7590760 polymorphisms with disease survivals in AML patients Kaplan-Meier evaluation of OS **A.** and DFS **B.** based on the rs2289195 genotypes in the AML cohort. Kaplan-Meier evaluation of OS **C.** and DFS **D.** based on the rs7346693 genotypes in the AML cohort; Kaplan-Meier evaluation of OS **E.** and DFS **F.** based on the rs1465825 genotypes in the AML cohort; Kaplan-Meier evaluation of OS **G.** and DFS **H.** based on the rs11695471 genotypes in the AML cohort. Kaplan-Meier evaluation of OS **I.** and DFS **J.** based on the rs2276598 genotypes in the AML cohort; Kaplan-Meier evaluation of OS **K.** and DFS **L.** based on the rs7590760 genotypes in the AML cohort. Patients failed to achieve CR were omitted in the DFS analysis.

Survival analysis showed that the six SNPs were significantly or in trend associated with prognosis of AML patients (Figure 3). Rs2289195 AA genotype showed significantly longer median OS and DFS versus GG/GA genotype group (*P=0.005* for OS; *P*=0.013 for DFS, Figure 3A, 3B); rs734693 TT genotype had significantly longer OS and marginally longer median DFS compared with CC/CT genotype (TT: 1119 days *vs* CT/CC: 529 days, *P*=0.033 for OS; TT: 1035 days *vs* CT/CC: 393 days, *P*=0.071 for DFS, Figure 3C, 3D); and rs2276598 CT/TT genotype had a marginally longer DFS compared with CC genotype (CT/TT: 494 days *vs* CC: 360 days, *P*=0.058, Figure 3J). While rs1465825 TT genotype showed a markedly longer median OS and a in trend longer median DFS versus TC/CC genotype (TT: 622 days *vs* TC/CC: 456 days, *P*=0.044 for OS; TT: 491 days *vs* TC/CC: 370 days, *P*=0.104 for DFS, Figure 3E, 3F); rs11695471 TT genotype showed in trend longer median OS and DFS versus TA/AA genotype (TT: 548 days *vs* TA/AA: 379 days, *P*=0.088 for OS, TT: 484 days *vs* TA/AA: 330 days, *P*=0.169 for DFS, Figure 3G, 3H); and rs7590760 GG/GC genotype had a in trend longer DFS compared with CC genotype (GG/GC: 469 days *vs* CC: 320 days, *P*=0.136, Figure 3L). Other SNPs (Supplementary Figure S2) showed no associated with median OS and DFS.

The characteristics of AML patients between genotype groups of *DNMT3A* rs11695471, rs2289195, rs734693, rs2276598, rs1465825 rs7590760 and other five SNPs were described (data not shown). The characteristics, such as gender, age, WBC count, BM blasts percentage, chemotherapy regimen, risk stratification or FAB classification at diagnosis showed no significant difference among the genotypes of all SNPs associated with OS or DFS.

### Multivariate analysis for disease prognosis

For the multivariate survival analysis, four well-established prognostic factors [27, 49–53]: age, WBC count, risk stratification and *DNMT3A* R882 mutation status were also considered for OS as these factors affected prognosis of AML significantly in univariate analysis (Figure 2). While WBC count, the risk stratification and *DNMT3A* R882 mutation status were also considered for DFS, because age was not significantly associated with DFS in univariate analysis (Figure 2B). It is noteworthy that the risk stratification was based on validated cytogenetics and molecular data, which included *FLT3*-ITD and *NPM1* mutation status in our study. Our results indicated that AML patients with *FLT3*-ITD mutation had a significantly shorter median survival than those without this mutation (*P*=0.002 for OS; *P*=0.023 for DFS, Figure S3A, S3B). *NPM1* mutated patients also had a significantly shorter median OS and DFS (*P*<0.05, Figure S3C, S3D). Results of multivariate survival analysis with individual *DNMT3A* SNPs were shown in Table 5. Rs2289195 G>A polymorphism showed as a favorable prognostic predictor independently for both OS and DFS (HR=0.434, 95% CI: 0.213-0.887, *P*=0.022 for OS; HR=0.468, 95% CI: 0.217-1.008, *P*=0.052 for DFS). Rs2276598 C>T polymorphism was an independent favorable predictor for DFS (HR=0.680, 95% CI: 0.480-0.963, *P*=0.030), while rs11695471 T>A polymorphism was an independent poor predictor for OS (HR=1.453, 95% CI: 1.010-2.093, *P*=0.044).

**Table 5:** Multivariate analysis of association between each individual *DNMT3A* SNPs and disease prognosis of AML patients

Endpoint	SNP	Model	N	HR (95% CI)	*P*^a^
**OS**	rs11695471	Dominant			
		TT	288	1.00 (reference)	
		TA/AA	56	1.453 (1.010-2.093)	**0.044**
	rs2289195	Recessive			
		GG/GA	316	1.00 (reference)	
		AA	27	0.434 (0.213-0.887)	**0.022**
	rs734693	Recessive			
		CC/CT	299	1.00 (reference)	
		TT	45	0.692 (0.436-1.098)	0.116
	rs2276598	Dominant			
		CC	147	1.00 (reference)	
		CT/TT	190	0.891 (0.664-1.194)	0.439
	rs1465825	Dominant			
		TT	137	1.00 (reference)	
		TC/CC	205	1.280 (0.951-1.724)	0.103
	rs7590760	Recessive			
		GG/GC	302	1.00 (reference)	
		CC	40	0.997 (0.645-1.540)	0.988
**DFS**	rs11695471	Dominant			
		TT	219	1.00 (reference)	
		TA/AA	36	1.484 (0.911-2.419)	0.111
	rs2289195	Recessive			
		GG/GA	231	1.00 (reference)	
		AA	24	0.468 (0.217-1.008)	**0.052**
	rs734693	Recessive			
		CC/CT	220	1.00 (reference)	
		TT	35	0.707 (0.410-1.221)	0.212
	rs2276598	Dominant			
		CC	107	1.00 (reference)	
		CT/TT	145	0.680 (0.480-0.963)	**0.030**
	rs1465825	Dominant			
		TT	105	1.00 (reference)	
		TC/CC	149	1.256 (0.881-1.792)	0.207
	rs7590760	Recessive			
		GG/GC	227	1.00 (reference)	
		CC	27	1.186 (0.693-2.033)	0.533

a Adjusted for age, WBC count, risk stratification and R882 mutation (OS); and for WBC count, risk stratification and R882 mutation (DFS).

Bold font indicates statistical significance.

**Abbreviations**: SNP, single nucleotide polymorphism; HR, hazard ratio; CI, confidence interval; OS, Overall survival; DFS, disease-free survival.

Furthermore, the prognostic significance of all 6 SNPs was analyzed simultaneously in a multivariate analysis model. After adjusting for age, WBC count, risk stratification and *DNMT3A* R882 mutational status, the results showed that only the rs2289195 G>A SNP had an independently favorable prognosis effect on OS; after adjusting by WBC count, risk stratification and *DNMT3A* R882 mutational status, both rs2289195 G>A and rs2276598 C>T polymorphisms appeared to be favorable prognostic predictors for DFS independently. However, other *DNMT3A* polymorphisms did not show association with prognosis indicated by either OS or DFS (Table 6).

**Table 6:** Multivariate analysis of association between *DNMT3A* polymorphisms and disease prognosis of AML patients

Endpoint	Variables in the model	HR	95% CI	*P*^a^
**OS**	**Age: >42 vs ≤42**	1.457	1.087-1.953	**0.012**
	**WBC: >14.43 vs ≤14.43**	1.328	0.987-1.788	0.061
	**Risk stratification**			
	**Low-risk *vs* Intermediate-risk**	0.807	0.494-1.320	0.393
	**High-risk *vs* Intermediate-risk**	2.067	1.509-2.831	**6.13×10^-6^**
	**R882 mutation: Yes vs No**	1.942	1.239-3.040	**0.004**
	**Rs2289195: AA vs GG/GA**	0.439	0.215-0.898	**0.024**
**DFS**	**WBC: >14.43 vs ≤14.43**	1.492	1.040-2.141	**0.030**
	**Risk stratification**			
	**Low-risk vs Intermediate-risk**	0.811	0.466-1.410	0.458
	**High-risk vs Intermediate-risk**	1.602	1.086-2.363	**0.017**
	**R882 mutation: Yes vs No**	2.066	1.174-3.636	**0.012**
	**Rs2276598: CT/TT vs CC**	0.657	0.463-0.933	**0.019**
	**Rs2289195: AA vs GG/GA**	0.450	0.208-0.970	**0.042**

**Abbreviations:** HR, hazard ratio; CI, confidence interval; OS, Overall survival; DFS, disease-free survival.

Bold font indicates statistical significance.

### A combined genotype analysis of rs11695471, rs2289195 and rs2276598 for AML survival

**Figure 4:**
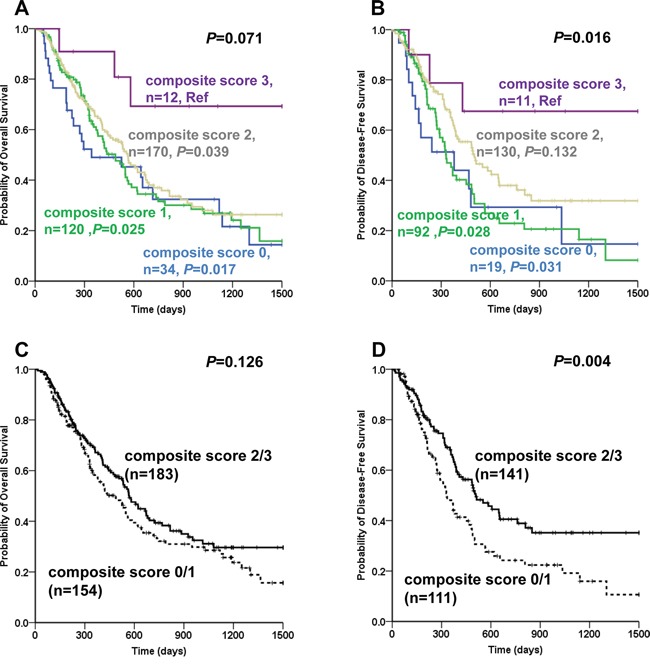
Combined genotype analysis of association between *DNMT3A* SNPs and disease prognosis in AML patients Kaplan-Meier evaluation of OS **A, C.** and DFS **B, D.** based on the composite score group of rs11695471, rs2289195 and rs2276598 in the entire AML cohort; Patients failed to achieve CR were omitted in the DFS analysis.

As rs11695471, rs2289195 and rs2276598 showed associations with AML prognosis individually, *DNMT3A* combined genotype score model was created by figuring up the genotype data of rs11695471, rs2289195 and rs2276598. Survival analysis of combined genotype showed that a defined favorable prognosis group (including composite score 2 plus composite score 3) showed a in trend longer median OS and a significantly longer median DFS than a defined unfavorable prognosis group (including composite score 0 plus composite score 1) (favorable prognosis group: 569 days *vs* unfavorable prognosis group: 488 days, *P*=0.126 for OS; favorable prognosis group: 508 days *vs* unfavorable prognosis group: 330 days, *P*=0.004 for DFS, Figure 4). After adjusting for age, WBC count, risk stratification and *DNMT3A* R882 mutational status (OS), and for WBC count, risk stratification and *DNMT3A* R882 mutational status (DFS), the results of multivariate survival analysis indicated that the unfavorable prognosis group (composite score 0 plus composite score 1) appeared to be an independent predicting factor for shorter OS and DFS (HR=1.346, 95% CI: 1.003-1.805, *P*=0.047 for OS; HR =1.717, 95% CI: 1.212-2.432, *P*=0.002 for DFS, Table 7).

**Table 7:** The potential association between *DNMT3A* genetic polymorphisms and AML prognosis by multivariate combined genotype analysis

Endpoint	Composite Score^a^	N	HR (95% CI)	*P*^b^
**OS**	composite score 3	12	1.00 (reference)	
	composite score 2	170	2.639 (0.828-8.418)	0.101
	composite score 1	120	3.218 (1.004-10.311)	**0.049**
	composite score 0	34	3.841 (1.152-12.806)	**0.028**
	composite score 2/3	183	1.00 (reference)	
	composite score 0/1	154	1.346 (1.003-1.805)	**0.047**
**DFS**	composite score 3	11	1.00 (reference)	
	composite score 2	130	1.984 (0.618-6.372)	0.250
	composite score 1	92	3.139 (0.974-10.112)	**0.055**
	composite score 0	19	3.854 (1.092-13.598)	**0.036**
	composite score 2/3	141	1.00 (reference)	
	composite score 0/1	111	1.717 (1.212-2.432)	**0.002**

**NOTE:** Bold font indicates statistical significance.

**Abbreviations:** SNP, single nucleotide polymorphism; HR, hazard ratio; CI, confidence interval; OS, Overall survival; DFS, disease-free survival.

a A DNMT3A combined genotype score model was created by figuring up the genotyped data of SNPs rs11695471, rs2276598 and rs2289195. Score 1 indicated favorable alleles (i.e. rs11695471 TT genotype, or rs2276598 CT/TT genotype, or rs2289195 AA genotype for prognosis) and score 0 indicated unfavorable alleles (i.e. rs11695471 TA/AA genotype, or rs2276598 CC genotype, or rs2289195 GG/GA genotype for prognosis). After adding up these scores, four score groups were generated: composite score 0, composite score 1, composite score 2 and composite score 3. Two prognosis groups were also defined: unfavorable prognosis (composite score 0/1) and favorable prognosis (composite score 2/3).

b Adjusted for age, WBC count, risk stratification and *DNMT3A* R882 mutation (OS); and for WBC count, risk stratification and *DNMT3A* R882 mutation (DFS).

### *DNMT3A* mutation/SNP may reduce *DNMT3A* mRNA expression in AML patients

**Figure 5:**
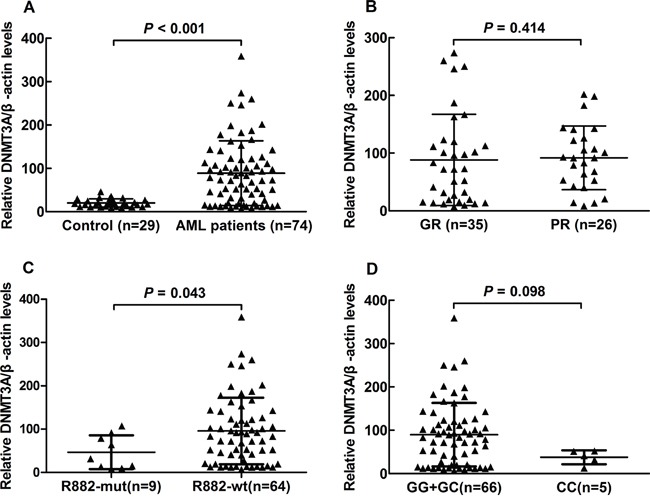
*DNMT3A* mRNA levels in AML patients with different CR status, R882 mutation status and rs7590760 genotypes **A.** Comparison of *DNMT3A* mRNA levels in AML patients and healthy volunteers. **B.** Comparison of *DNMT3A* mRNA levels in the AML patients with good response (GR) or poor response (PR). **C.** Comparison of *DNMT3A* mRNA levels in AML patients with or without R882 mutation. **D.** Comparison of *DNMT3A* mRNA levels in AML patients with rs7590760 GG/GC genotype and rs7590760 CC genotype. Each triangle represents one patient and the number of patients in each group is shown. Horizontal lines indicate the mean *DNMT3A*/β-actin ratio. Relative expression levels in each patient were determined in duplicates and normalized to β-actin. Relative expression was calculated by 2^−ΔCt^.

*DNMT3A* mRNA levels were detected using semi-quantitative reverse-transcriptase PCR method in PBMCs from 29 healthy volunteers and 74 unselected de novo AML patient samples with known CR status, *DNMT3A* mutation (R882 mutation, n=9) and *DNMT3A* SNP status. As shown in Figure 5A, PBMCs *DNMT3A* mRNA levels were markedly higher in AML patients than those in healthy volunteers (*P*<0.001). In order to assess potential influence of *DNMT3A* mRNA levels on complete remission (CR), 61 AML patients were divided into good response (GR) group and poor response (PR) group. As shown in Figure 5B, no difference in PBMCs *DNMT3A* mRNA levels was observed between GR and PR patients (*P*=0.414). Mean *DNMT3A* mRNA levels in *DNMT3A* R882 wildtype AML patients (n=64) was 2.06 times that of patients with R882 mutation (n=9, *P*=0.043, Figure 5C). Mean *DNMT3A* mRNA levels in patients with rs7590760 GG/GC genotype (n=66) was 2.38 times that of rs7590760 CC genotype (n=5, *P*=0.098, Figure 5D). No difference in *DNMT3A* mRNA expression among genotypes of other *DNMT3A* polymorphisms was observed in AML patients (data not shown).

### Lower *DNMT3A* levels predicted an inferior prognosis in AML patients from a TCGA dataset

**Figure 6:**
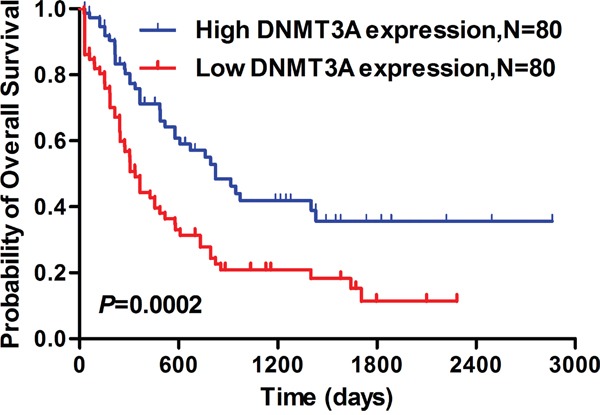
Lower *DNMT3A* mRNA expression predicted significantly shorter median overall survival in AML patients Kaplan-Meier comparison of overall survival in AML patients with low *DNMT3A* expression and high *DNMT3A* expression.

Survival analysis from a TCGA data set (seen in PATIENTS AND METHODS) found that the AML patients with lower *DNMT3A* expression showed significantly shorter median OS than those with higher *DNMT3A* expression (lower *DNMT3A*: 335 days *vs* higher *DNMT3A*: 822 days, *P*<0.001, Figure 6).

To sum up, our results indicated that rs11695471, rs734693 and rs2276598 were significantly associated with chemosensitivity in AML patients, while rs11695471, rs2289195, and rs2276598 can independently predict AML patients' disease prognosis.

## DISCUSSION

To our knowledge, this study firstly identified a strong association between *DNMT3A* genetic polymorphisms and chemosensitivity and prognosis in AML patients. We observed that rs11695471, rs734693 and rs2276598 were significantly associated with chemosensitivity, while rs11695471, rs2289195 and rs2276598 had significant associations with the prognosis (OS or DFS) in AML patients. Further, *DNMT3A* combined genotype score model (created by figuring up data from rs11695471, rs734693 and rs2276598 genotypes for chemosensitivity; rs11695471, rs2289195, rs2276598 genotypes for prognosis) also showed similar results in our study population. In agreement with previous investigations [9, 11, 21, 23, 27], we confirmed that *DNMT3A* R882 mutations predicted a significantly poor prognosis in our AML patients.

For the multivariate survival analysis, the prognostic effect of *DNMT3A* SNPs was adjusted by four well-established prognostic factors including age, WBC count, risk stratification of disease and *DNMT3A* R882 mutation status. In our study, *FLT3*-ITD and *NPM1* mutations were considered in the risk stratification according to NCCN Guidelines (Version 1.2015 Acute Myeloid Leukemia), we included risk stratification but not mutational status of *FLT3*-ITD and *NPM1* as individual variables in our multivariate analysis. In agreement with previous reports [54–56], *FLT3*-ITD predicted a significantly poor prognosis in our AML patients. However, patients positive for *NPM1* mutation had a significantly shorter median survival, though some researchers reported that *NPM1* mutation conferred a favorable prognosis in AML patients with younger age or normal karyotype [57, 58]. This disagreement may be explained by the significant co-occurrence of *NPM1* and *FLT3*-ITD mutations, and prognosis of patients carrying both mutations was similar with that of patients with *FLT3*-ITD mutation alone [57–59]. In consistent with previous reports [23, 60, 61], we observed that *NPM1* mutations occurred concurrently with *FLT3*-ITD and *DNMT3A* R882 mutation in our samples. Besides, Boissel N et al [62] showed no significant difference in AML prognosis between *NPM*-mutated and -nonmutated patients with normal karyotype.

Hayette S et al [63] reported that *DNMT3B* overexpression may serve as a poor prognostic marker in AML, and *DNMT3B* expression was inversely correlated with *DNMT3A* level. Therefore, wondering whether the outcome effect of *DNMT3A* mutation/SNP on AML could act through affecting *DNMT3A* mRNA expression, we determined *DNMT3A* transcript levels in PBMCs from de novo AML patients. Consistent with previous reports [64, 65], we observed that AML patients showed significantly increased *DNMT3A* mRNA level than healthy volunteers, which indicated that high *DNMT3A* expression might contribute to the pathogenesis of leukemia [64]. Though evidence has shown that *DNMT3A* mutation can reduce the enzyme activity [9, 11, 66, 67], influence of R882 mutation on *DNMT3A* mRNA expression has not been described yet. Here we found that AML patients with R882 mutation showed significantly lower *DNMT3A* mRNA level. Fortunately, homozygote mutation of rs7590760 G>C polymorphism which could predicted a trend of shorter OS and DFS in AML, also showed a trend of decreased *DNMT3A* mRNA levels in our study. Furthermore, survival analysis indicated that lower *DNMT3A* expression displayed significantly shorter median OS in AML patients with data from a TCGA data set.

Although there is no report regarding association between *DNMT3A* polymorphisms and the outcome of AML patients, we speculate the possible mechanisms underline association between *DNMT3A* SNPs and chemosensitivity or disease prognosis may be explained by change in *DNMT3A* mRNA expression. Herein we found that AML patients positive for R882 mutation showed significantly lower *DNMT3A* mRNA levels. And also, rs7590760 G>C rare homozygotes presented a trend of decreased *DNMT3A* mRNA levels as compared to carriers of the rs7590760 G wildtype allele. In our study, the significant SNPs rs11695471, rs2289195, rs734693, rs2276598, rs1465825 and rs7590760 in univariate analysis were located in introns or synonymous, pair-wise LD analysis showed that r^2^ ranges from 0 to 0.63 for these SNPs. For the three significant SNPs (rs11695471, rs2289195 and rs2276598) associated with AML prognosis in multivariate analysis, the r^2^ ranges from 0.02 to 0.04. Therefore, the positive association may not be explained by LD between these SNPs (Figure 1). Of note, the 6 significant SNPs appeared to cluster at 3′ region of the gene. Evidence has shown that variants in 3′ UTR may change miRNAs targeting and subsequent change in mRNA expression. For instance, Cheng et al [68]reported that a polymorphic nucleotide T deletion (rs34351976) in *NPM1* 3′UTR could lead to the formation of an illegitimate miR-337-5p binding site and thus predicted an adverse prognosis in AML. Similarly, there may exist SNP(s) located in *DNMT3A* 3′-UTR which were in high LD with the positively associated SNPs. Of course, additional studies are required to determine the functional SNP(s) at the locus.

Certainly, several limitations of our study are unneglectable. First, though risk factors such as gender, age, blood routine indexes (clinical factors), karyotype, risk stratification (cytogenetics factor) and somatic mutations (including *FLT3-ITD*, *NPM1* and *DNMT3A*) were adjusted during data analysis, other important risk factors, such as *CEBPA*, *KIT* and *IDH1/2* mutations, which may also contribute to the prognosis of AML [22, 69–72], were ignored in this study. Second, the number of samples for mutant homozygote of some *DNMT3A* SNPs was relatively small because of the low MAF values, which may have led to limited statistical power. Third, only the genetic model (dominant or recessive model) which had statistical significance was chosen to evaluate the association between each SNP and AML outcomes in our study. Finally, this study required confirmation in independent studies with more sufficient power to detect single and combined genotype effects of *DNMT3A* genetic polymorphisms.

In summary, the current study firstly indicated that (i) there was strong association between *DNMT3A* genetic polymorphisms and the treatment outcomes in AML patients, and (ii) an available score model evaluating multiple polymorphisms in *DNMT3A* could predict chemosensitivity or prognosis in AML patients, and (iii) especially, a strategy estimating multiple noncoding polymorphisms in epigenetic modification related genes, such as *DNMT3A*, could predict the treatment outcomes in AML patients, which may improve rational design for more precise therapeutic regimens and prognostic stratification.

## PATIENTS AND METHODS

### Patients and treatment

A prospective analysis of 352 Chinese Han AML patients with available whole peripheral blood or bone marrow samples who hospitalized in Xiangya Hospital of Central South University between June 2011 and July 2015 was evaluated. FAB classification was applied to diagnose AML patients in our study [73]. Patients with the FAB-M3 subtype were excluded because of the occurrence of the specific fusion gene PML-RARa caused by translocation t(15;17) which makes the treatment effect better than other types of AML. And patients with serious diseases or other cancers, therapy-related AML or secondary AML arising from a prior myelodysplastic syndrome were also excluded. Clinical study admission (registration number: ChiCTR-PPC-14005297) was approved. Informed consent has been obtained for this study, which was approved by the Ethics Committee of Institute of Clinical Pharmacology of Central South University (registerNo.CTXY-120025-2) and was implemented in strict accordance with the Declaration of Helsinki. All patients received standard induction chemotherapy consisted of recommended dose anthracyclines for three consecutive days and 100-200 mg/m^2^ cytarabine for seven consecutive days. Genomic DNA for polymorphism analyses was available for 344 cases from this cohort. Once CR was achieved, the patients were treated with hematopoietic cell transplantation (HCT) or cycles of high-dose cytarabine, followed by periods of consolidation chemotherapy consisted of anthracyclines and cytarabine.

### Definition and end points

Major end points were defined as described previously [50, 74, 75]. Complete remission (CR) following one or two cycles of induction chemotherapy was used to evaluate chemosensitivity. CR was characterized by < 5% bone marrow (BM) blast cells and the absolute counts of neutrophils >1 × 10^9^/L and platelets >100 × 10^9^/L in addition to no evidence of extramedullary leukemia. Partial remission was characterized by: achieving all criteria of CR in hematology; BM blasts declining to 5% to 25% or to 5% if Auer rod positive. In this study, chemosensitivity groups were defined: the relatively good response (GR) group (including receiving CR or partial remission) and the relatively poor response (PR) group (except for CR or partial remission). Disease-free survival (DFS) was equal to the time interval from the first CR to relapse or death for any cause. Overall survival (OS) was equal to the time length from diagnosis to death for any cause. For patients without relapse or death event by the end of the study follow-up, survival end points were censored by the last follow-up. Patients failed to follow-up were excluded in the study. The risk stratification including *FLT3*-ITD status and *NPM1* mutation status based on validated cytogenetics and molecular data was classified according to NCCN Guidelines Version 1.2015 Acute Myeloid Leukemia [76].

### *FLT3*-ITD and PCR-gel-electrophoresis

The internal tandem duplications of *FLT3* (*FLT3*-ITD) were tested as described [55, 77]. Briefly, the fragment from exon 14 to exon 15 of *FLT3* were amplified using previously described primers (Supplementary Table S1) [78] from genomic DNA by the following steps: denaturing at 94°C for 45 seconds, annealing at 57°C for 45 seconds, and extension at, 72°C for 30 seconds, for 35 cycles on a Master cycler® pro (Eppendorf, Germany) including a pre-denaturation at 94°C and a post-extension at 72°C for 5 minutes, respectively. PCR products were electrophoresed on 2% agarose gels and visualized under UV light with Gel Red (red) staining. A length of 329 base pair (bp) was produced from wild-type alleles. PCR bands larger than 329 bp were seen as *FLT3*-ITD. Repeat analyses were done on samples with additional bands.

### Detection of *DNMT3A* R882 and *NPM1* mutations by PCR-pyrosequencing

Detection of *DNMT3A* R882 (exon 23) and *NPM1* (exon 12) mutations was carried out by PCR and analyzed with Pyro Mark Q24 Advance software (Qiagen, Germany) similarly as previous described [79–81]. DNA separated from whole peripheral blood or bone marrow of patients was amplified with biotinylated primer to harvest single-stranded templates for pyrosequencing. PCR was performed on a Master cycler® pro (Eppendorf, Germany). Next, the single-stranded templates were analyzed using Pyro Mark Q24 station (Qiagen, Germany) according to instrument instructions. Pyrosequencing assay involves 3 primers: forward, reverse and sequencing (Supplementary Table S1). Necessarily, Sanger sequencing was also applied to verify the reliability of the pyrosequencing result. The results displayed that two R882 variants (R882H and R882C) could be detected simultaneously, and the results of pyrosequencing and Sanger sequencing were consistent completely.

### SNPs selection

The full sequence of the human *DNMT3A* gene investigated in this study included all exons and introns (109.63 kb), which were pinpointed to chromosome 2, position 25232961-25342590 (www.ncbi.nlm.nih.gov/genbank/). Genetic variation data for the entire *DNMT3A* gene were obtained from the 1000 Genomes Project (http://www.1000genomes.org/) for 105 Han Chinese individuals from Southern China (CHS). From this database, a total of 300 SNPs in *DNMT3A* have been identified in the CHS population, and 121 were common SNPs with minor allele frequencies (MAF) ≥0.05. We identify 44 SNPs (tagging SNPs) that efficiently tags 112 of the SNPs using Haploview, version 4.2 (Broad Institute of MIT and Harvard, Cambridge, M A, USA), a software package that provides computation of linkage disequilibrium (LD) statistics [82]. r2>0.8 indicates a strong LD between two pairwise SNPs. Based on consulting literature material, we selected 15 tagging SNPs that best represented potential functional SNPs (priority: preferring missense to synonymous codon to intron variant). Finally, we successfully genotyped 10 tagging SNPs using A Mass ARRAY system from Sequenom (Supplementary Figure S1). *DNMT3A* SNP (rs7560488) was also selected according to the publication by Wu H et al [31], which showed an increased risk of gastric cancer for the C allele (variant) by modulating promoter activity in Chinese population.

### Genotyping of *DNMT3A* polymorphisms

For *DNMT3A* genotyping, whole peripheral blood genomic DNA was extracted from AML patients using a E.Z.N.A.^®^ SQ Blood DNA Kit II (Omega Bio-Tek, USA). A Mass ARRAY system from Sequenom was used to genotype *DNMT3A* 10 SNPs. The assays were validated by Polymerase chain reaction–restriction fragment length polymorphism (PCR-RFLP) or by Sanger sequencing (ABI 3730XL DNA Analyzer, Applied Biosystems, USA) or by pyrosequencing methods for 5% of the samples, and the results were 100% concordant. *DNMT3A* rs7560488 was genotyped by Sanger sequencing. The forward and reverse primer pairs of PCR, and the following extending primer or sequencing primer were shown in Supplementary Table S1.

### *DNMT3A* combined genotype score model

A *DNMT3A* combined genotype score model was created by figuring up the genotyped data of multi-SNPs. Score 1 indicated favorable genotype(s) (i.e. rs11695471 TT genotype, or rs734693 CC genotype, or rs2276598 CT/TT genotype for chemosensitivity; rs2289195 AA genotype, or rs11695471 TT genotype, or rs2276598 CT/TT genotype for prognosis) and score 0 indicated unfavorable genotype(s) (i.e. rs11695471 TA/AA genotype, or rs734693 CT/TT genotype, or rs2276598 CC genotype for chemosensitivity; rs2289195 GG/GA genotype, or rs11695471 TA/AA genotype, or rs2276598 CC genotype for prognosis). After adding up the score of every SNP, four score groups were generated: composite score 0, composite score 1, composite score 2 and composite score 3. And then another two chemosensitivity groups were defined: the relatively unfavorable response group (composite score 0) and the relatively favorable response group (including composite score 1, composite score 2 and composite score 3). Two prognosis groups were also defined: the relatively unfavorable prognosis group (including composite score 0 and composite score 1) and the relatively favorable prognosis group (including composite score 2 and composite score 3).

### Analysis of *DNMT3A* mRNA expression

We unbiasedly selected 74 cases' bone marrow (BM) or peripheral blood mononuclear cells (PBMCs) from this cohort, whose baseline characteristics were shown in Supplementary Table S3. *DNMT3A* mRNA expression was also assessed by RT-qPCR and compared with 29 normal peripheral blood samples obtained from healthy donors who had no prior history of malignancy. Mononuclear cells were separated by centrifuging (400 × g) and layering for 30 minutes at 18°C∼25°C mixing equal volume of Histopaque-1077 (Sigma–Aldrich, St. Louis, MO). Total RNA was purified with RNAiso Plus reagents (Takara-Bio, Dalian, China) and stored at −80 °C until analysis. The cDNA was reverse transcribed using Prime Script™ RT reagent kit (Takara-Bio, Dalian, China). QRT-PCR was performed on a LC480® real-time PCR system (Roche, USA) using the real-time PCR quant kit (Takara, Biotechnology, Dalian, China). The forward primer of *DNMT3A* was 5′-AGTAC GACGACGA CGGCTA-3′, the reverse primer was 5′-CACACTCCACGCAAAAGCAC-3′. The β-actin mRNA expression was used as an endogenous control.

### *DNMT3A* expression Data extract from TCGA database

A dataset including the information on *DNMT3A* mRNA expression and overall survival (OS) of AML (n=173 for LAML gene expression, pancan normalized) was obtained from the UCSC Cancer Genomics Browser of TCGA (https://genome-cancer.soe.ucsc.edu/). *DNMT3A* expression was divided into lower *DNMT3A* expression group and higher *DNMT3A* expression group according to a cut-off value of median *DNMT3A* expression. Then, we evaluated the association of *DNMT3A* expression with overall survival (OS) in this AML cohort. Thirteen samples were not included in survival analysis due to lacking of survival information.

### Statistical analysis

Chi-square (χ^2^) test was used for determining whether *DNMT3A* 11 SNPs agreed with Hardy–Weinberg equilibrium. The linkage disequilibrium (LD) of genotypes was analyzed by Haploview software (available at http://www.broad.mit.edu/mpg/haploview). The frequencies of the genotypes were compared using χ^2^ test or Fisher's test in baseline' characteristics of patients or status of achieving CR after one or two cycles of induction chemotherapy. A logistic regression model was used to ascertain independent predictive factors for chemosensitivity, including gender, age, FAB classification, rs11695471, rs2276598 and rs734693. The OR and 95% CI indicated the relative risk level. Comparisons of continuous variables between or among genotype groups were performed using nonparametric tests. The significance of observed differences in proportions was tested by the χ^2^ test and Fisher's exact test when data were spare. The primary analysis was performed on OS. Sensitivity analyses were conducted on CR rate and DFS. The median OS and DFS evaluations were performed by Kaplan-Meier curve. Cox's proportional hazard model was used to identify independent prognostic predictors for OS and DFS using a backward conditional procedure. The following variables were included for the analyses: rs11695471, rs2289195, rs7346693, rs2276598, rs1465825 and rs7590760, age, WBC count, risk stratification and *DNMT3A* R882 mutational status. The HR and 95% CI indicated the relative risk level for survivals. Two-sided *P* <0.05 represented a statistical significance of difference. SPSS 18.0 was used for statistical analyses.

## SUPPLEMENTARY FIGURES AND TABLES




