# Supplementary material for: Evaluation of DNMT3A genetic polymorphisms as outcome predictors in AML patients
Source: Oncotarget. 2016 Aug 9;7(37):60555–74. doi: 10.18632/oncotarget.11143 (PMC5312402; doi:10.18632/oncotarget.11143)
Supplement: Supplementary file 1 [file oncotarget-07-60555-s001.pdf]

Evaluation of *DNMT3A* genetic polymorphisms as outcome predictors in AML patients

SUPPLEMENTARY FIGURES AND TABLES

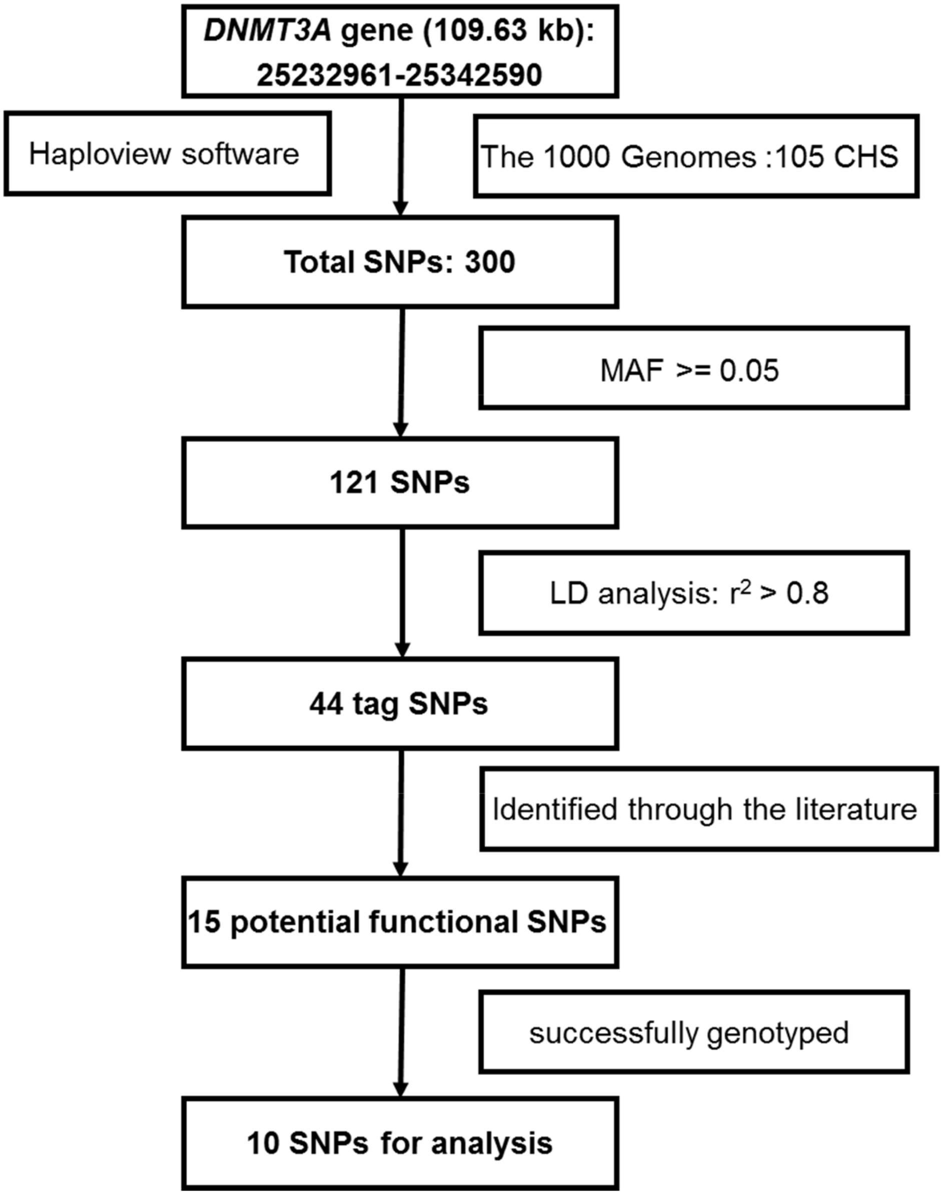

Supplementary Figure S1: Flow diagram for SNPs selection in the study. CHS, Han Chinese individuals from Southern China; MAF, minor allele frequency; LD, linkage disequilibrium.

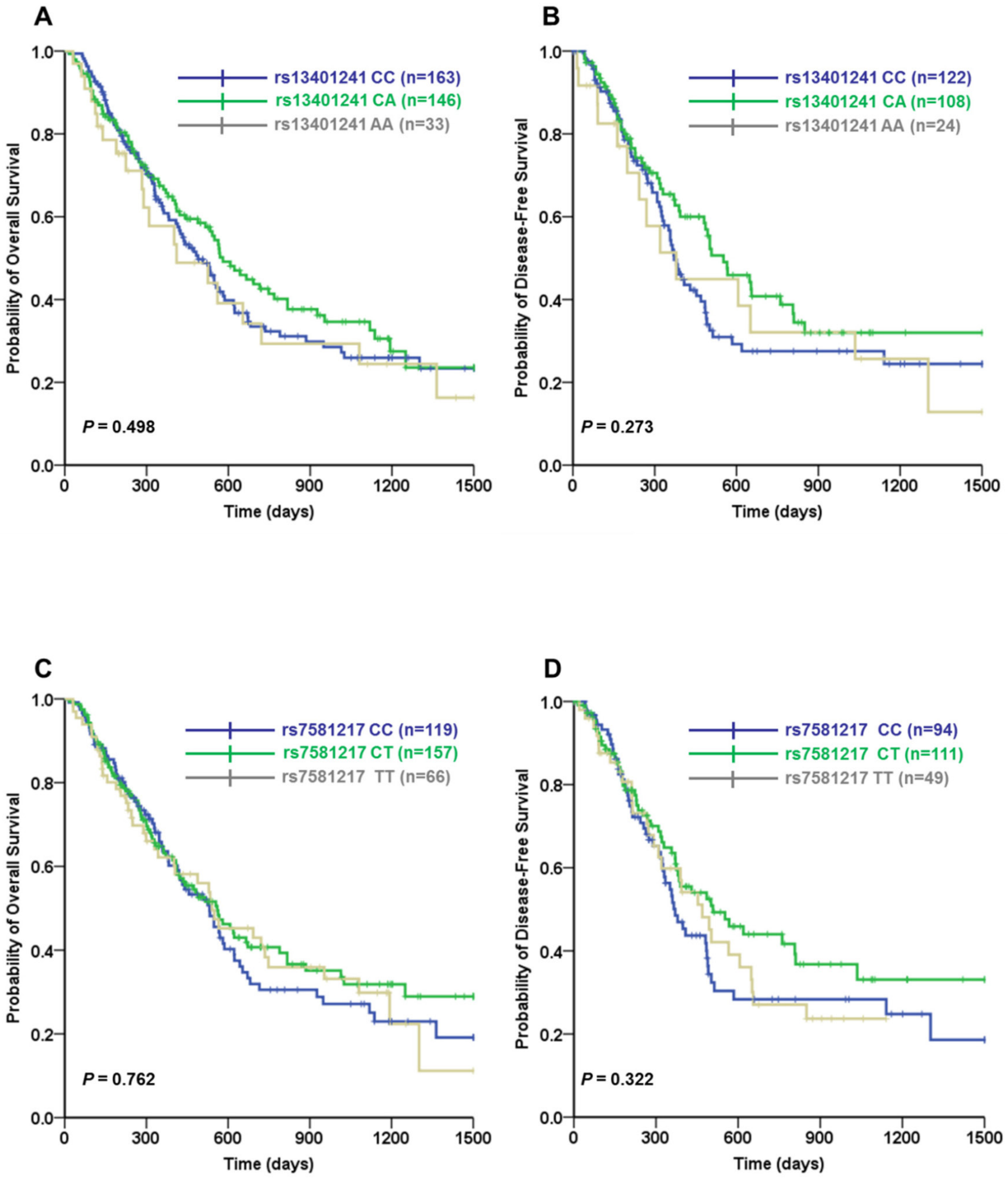

Supplementary Figure S2: Associations of rs13401241, rs7581217, rs749131, rs41284843 and rs7560488 with disease survivals in AML patients. Kaplan-Meier evaluation of OS **A.** and DFS **B.** based on the rs13401241 genotypes in the AML patients. Kaplan-Meier evaluation of OS **C.** and DFS **D.** based on the rs7581217 genotypes in the AML patients; Kaplan-Meier evaluation of OS

(*Continued*)

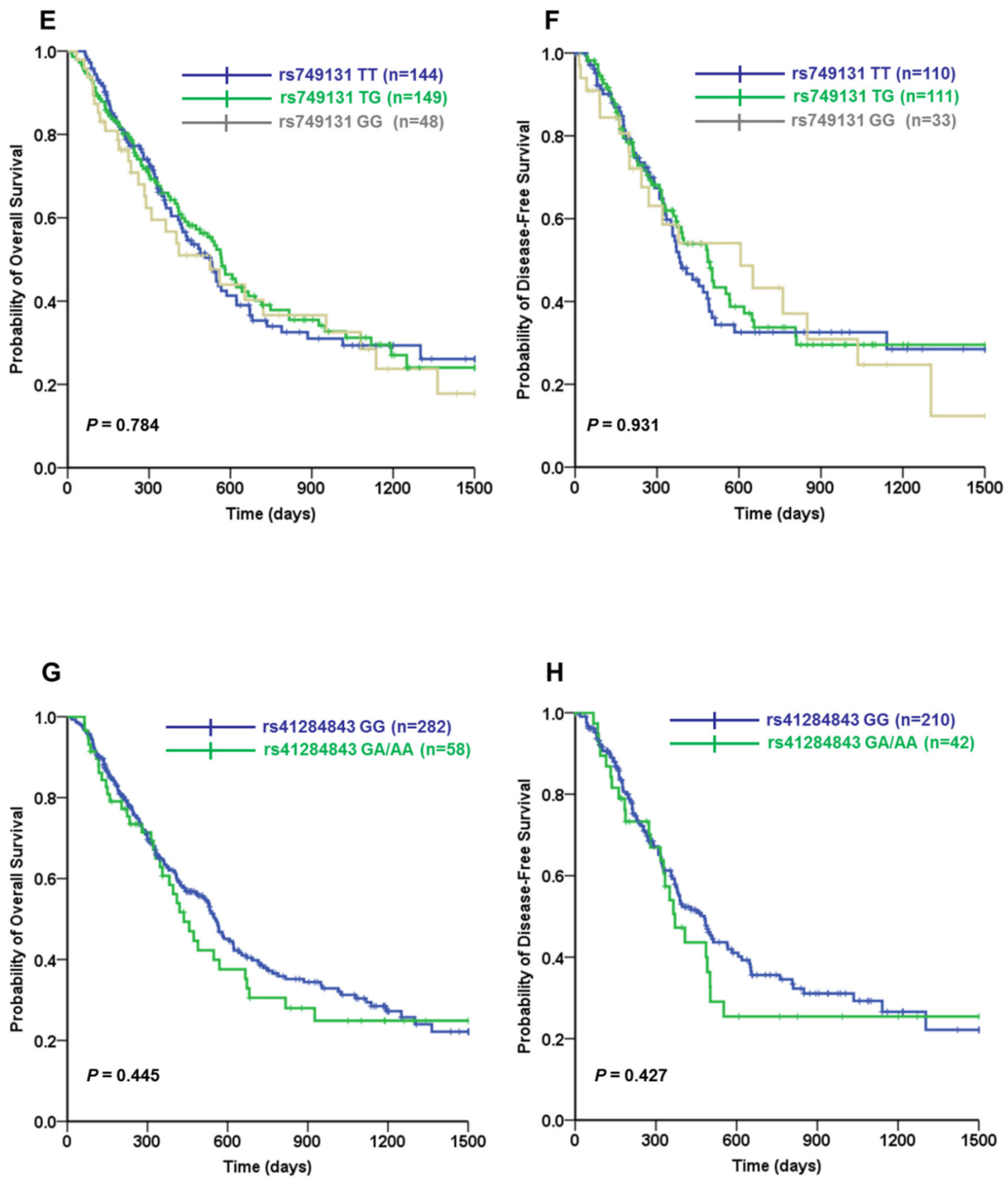

Supplementary Figure S2 (*Continued*): E. and DFS **F.** based on the rs749131 genotypes in the AML patients; Kaplan-Meier evaluation of OS **G.** and DFS **H.** based on the rs41284843 genotypes in the AML patients. Kaplan-Meier evaluation of OS

(*Continued*)

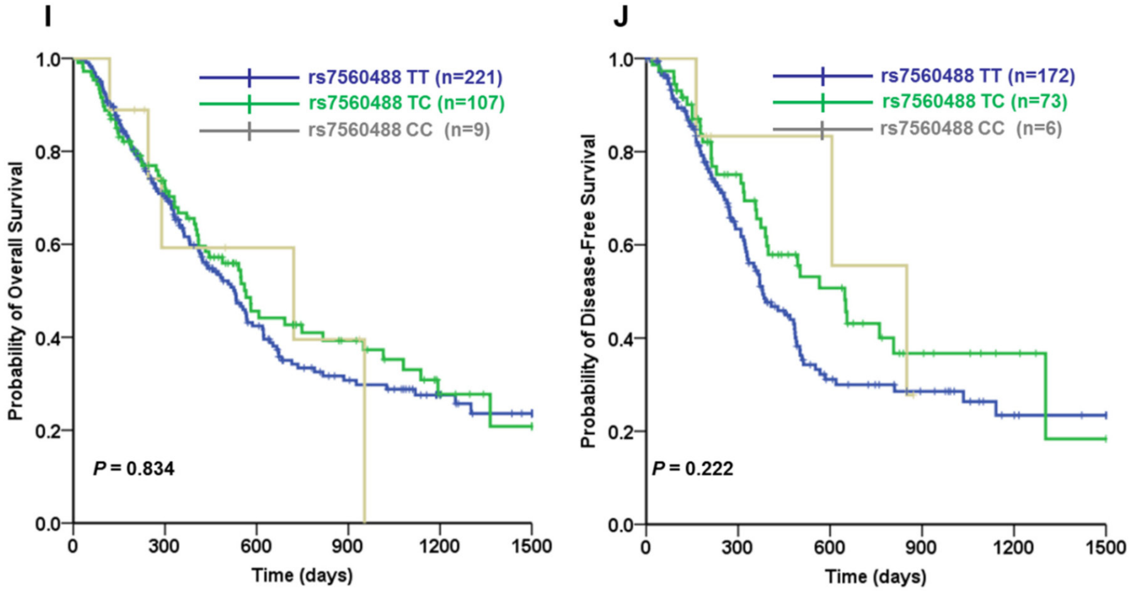

Supplementary Figure S2 (*Continued*): I. and DFS **J.** based on the rs7560488 genotypes in the AML patients. Patients failed to achieve CR were omitted in the DFS analysis.

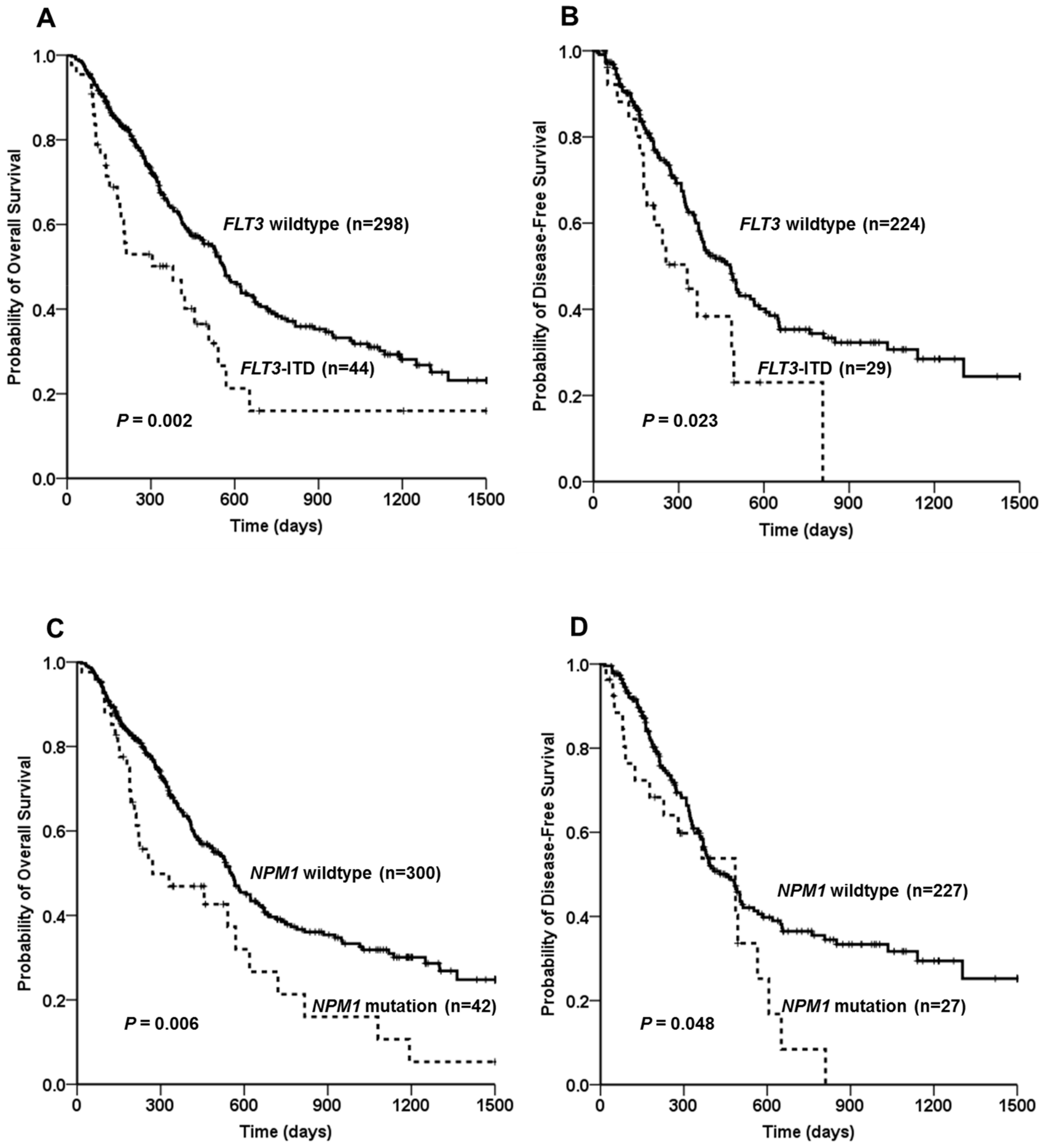

Supplementary Figure S3: Associations of FLT3-ITD and NPM1 mutation with disease survivals in entire AML patients. Kaplan-Meier evaluation of OS **A.** and DFS **B.** based on the status of *FLT3*-ITD in the entire AML cohort. Kaplan-Meier evaluation of OS **C.** and DFS **D.** based on the status of *NPM1* mutation in the entire AML cohort. Patients failed to achieve CR were omitted in the DFS analysis.

Supplementary Table S1: Primer sequences of *FLT3*-ITD, *NPM1* mutation, *DNMT3A* R882 mutation and *DNMT3A* SNPs analysis

SNP	Forward primer	Reverse primer	Extending or sequencing primer
***FLT3*-ITD**	GCAATTTAGGTA TGAAAGCCAGC	CTTTCAGCATTTT GACGGCAACC	
***NPM1* mut**[a]	TTTTTTTCCAGGCTA TTCAAGATC	GGGAAAGTTCTCA CTCTGCATTAT	TTAAAGAGACT TCCTCCAC
**R882 mut**[a]	GGTCCTGCTGTGT GGTTAGACG	AAGAGGTGGCGGATGACTG	CTCTGCCTCGCCAAG
**rs11695471**[b]	ACGTTGGATGAGGAGTTCG GCTCCTTTTTC	ACGTTGGATGGGAGT CTGCATGTAACGAAC	AACACGTAG CCCTAGCA
**rs2289195**[b]	ACGTTGGATGACGGTAG GTACCATCCTGTC	ACGTTGGATGGGGCAG AAATATCCAAGGAG	AGGTTCGGAAGC ACCAGCTGAGAA
**rs734693**[b]	ACGTTGGATGTAAGA CACTCAGCATGCCAG	ACGTTGGATGAACTAGTA TCACCCAGGCAG	GCCAATGGGAAGGTG ACCCAAGTGGA
**rs2276598**[b]	ACGTTGGATGCCATGATTGAAT GGGCCCTG	ACGTTGGATGGTGCCCT CATTTACCTTCTG	ACCTTCTGGTGGCTC
**rs1465825**[b]	ACGTTGGATGTGGGCA GACAACAGTGATCC	ACGTTGGATGAATACA GGATCAGGGCAGAG	CGATGTATTTTTG TCGGGCTTA
**rs7590760**[b]	ACGTTGGATGGACTGG AAGACATTCCTCAC	ACGTTGGATGCATCATCT CCTTCAAGGGTG	TGCAAAAGGTCAGGTA
**rs13401241**	ACGTTGGATGTTTGGCTACT GGGTACCTAC	ACGTTGGATGCCACAC ACTTTTTCCAGCAG	CCCCTGAATTCACAT ATTAACTGA
**rs7581217**[b]	ACGTTGGATGCCATG TGCATGTCTTTGGAG	ACGTTGGATGATCCTCC AGCTGAGGCATGT	GCTTCAGGGCAGACCATCA
**rs749131**[b]	ACGTTGGATGATCCAG TACACAGTAGGTGC	ACGTTGGATGACCTCTGAG GTCTCTATGTC	GGAGGGCCAGACA CAGGGGTGCACT
**rs41284843**[b]	ACGTTGGATGAGATGC CCGCCATGCCCTC	ACGTTGGATGTGCTCACCT TTCGGTCCTC	CCTAAGAGCTGCT GGTGTCCCC
**rs7560488**[c]	AGGCAGACACAAAT GCATAAAT	GTCATAAGTACAA CCACCACCG	GTCATAAGTACAACC ACCACCG

[a]Primer sequences of *NPM1* mutation and *DNMT3A* R882 mutation analyzed by pyrosequencing.
[b]The primer sequence of *DNMT3A* 10 SNPs genotyped by Mass ARRAY® System.
[c]The primer sequence of *DNMT3A* rs7560488 genotyped by Sanger sequencing.
Abbreviations: SNP, single nucleotide polymorphism; R882 mut, *DNMT3A* R882 mutation.

Supplementary Table S2: Distribution of *FLT3*-ITD and *DNMT3A* R882 genotypes according to *NPM1* mutational status

Concurrent mutations	*NPM1* mutation, n=42	*NPM1* wildtype, n=300	*P*
	No. of patients (%)	No. of patients (%)	
***FLT3*-ITD**			**1.51E-6**
**Positive**	15 (36.6)	29 (9.7)	
**Negative**	26(63.4)	270 (90.3)	
***DNMT3A* R882 mutation**			**5.15E-4**
**Positive**	9 (21.4)	18 (6.0)	
**Negative**	33 (78.6)	282 (94.0)	

NOTE: Bold font indicates statistical significance. *FLT3*-ITD analysis was failed in 2 patients.

Supplementary Table S3: Comparison of characteristics of 344 AML patients for outcome analysis and 74 AML patients for *DNMT3A* mRNA detection

See Supplementary File 1
