# Supplementary material for: Evaluation of DNMT3A genetic polymorphisms as outcome predictors in AML patients
Source: Oncotarget. 2016 Aug 9;7(37):60555–74. doi: 10.18632/oncotarget.11143 (PMC5312402; doi:10.18632/oncotarget.11143)
Supplement: Supplementary file 2 [file oncotarget-07-60555-s002.docx]

**Table S3: Comparison of characteristics of 344 AML patients for outcome analysis and 74 AML patients for *DNMT3A* mRNA detection.**

| **Characteristic** |  | **344 Patients** |  | **74 Patients** |  | ***P*** |
| --- | --- | --- | --- | --- | --- | --- |
| **Sex** |  |  |  |  |  | 0.238 |
| **Male** |  | 188(54.7) |  | 46(62.2) |  |  |
| **Female** |  | 156(45.3) |  | 28(37.8) |  |  |
| **Age, years** |  | 42(14-79) |  | 44(14-79) |  | 0.260 |
| **BSA** |  | 1.6(1.2-2.1) |  | 1.6(1.3-2.0) |  | 0.202 |
| **BMI** |  | 22.4 (15.6-35.1) |  | 22.0(17.6-31.5) |  | 0.434 |
| **FAB classification** |  |  |  |  |  | 0.763 |
| **M0** |  | 1(0.3) |  | 0(0.0) |  |  |
| **M1** |  | 20(5.8) |  | 3(4.1) |  |  |
| **M2** |  | 165(48.0) |  | 32(43.2) |  |  |
| **M4** |  | 50(14.5) |  | 16(21.6) |  |  |
| **M5** |  | 63(18.3) |  | 16(21.6) |  |  |
| **M6** |  | 5(1.5) |  | 0(0.0) |  |  |
| **M7** |  | 1(0.3) |  | 0(0.0) |  |  |
| **Other AML** |  | 37(10.8) |  | 7(9.5) |  |  |
| **Mix(AML+ALL)** |  | 2(0.6) |  | 0(0.0) |  |  |
| **Risk stratifications** |  |  |  |  |  | 0.205 |
| **Low risk** |  | 48(14.0) |  | 16(21.6) |  |  |
| **Intermediate risk** |  | 196(57.0) |  | 41(55.4) |  |  |
| **High risk** |  | 100(29.1) |  | 17(23.0) |  |  |
| ***DNMT3A* R882 mutation** |  |  |  |  |  | 0.749 |
| **Positive** |  | 27(7.8) |  | 5(6.8) |  |  |
| **Negative** |  | 317(92.2) |  | 69(93.2) |  |  |
| ***FLT3*-ITD** |  |  |  |  |  | 0.294 |
| **Positive** |  | 44(12.8) |  | 12(17.6) |  |  |
| **Negative** |  | 298(86.6) |  | 56(82.4) |  |  |
| **Chemotherapy regimens** |  |  |  |  |  | 0.664 |
| **Mitoxantrone + AraC** |  | 88(25.6) |  | 20(29.4) |  |  |
| **AraC + ACLA + G-CSF** |  | 107(31.1) |  | 24(35.3) |  |  |
| **Daunorubicin + AraC** |  | 42(12.2) |  | 4(5.9) |  |  |
| **Idarubicin + AraC** |  | 47(13.7) |  | 9(13.2) |  |  |
| **THP + AraC** |  | 29(8.4) |  | 4(5.9) |  |  |
| **Others** |  | 31(9.0) |  | 7(10.3) |  |  |
| **BM blasts, %** |  | 70.0(2.0-99.0) |  | 54.0(2.0-96.0) |  | 0.053 |
| **PB blasts, %** |  | 60.0(6.0-97.0) |  | 66.0(8.0-96.0) |  | 0.464 |
| **WBC, ×10^9^/L** |  | 14.5(0.5-436.2) |  | 13.5(0.5-436.2) |  | 0.844 |
| **RBC, ×10^12^/L** |  | 2.2(0.6-4.9) |  | 2.2(1.0-4.4) |  | 0.950 |
| **Hemoglobin, g/L** |  | 71.0(27.0-155.0) |  | 75.0(38.0-128.0) |  | 0.828 |
| **Platelets, 10^9^/L** |  | 30.0(3.0-546.0) |  | 34.0(5.0-586.0) |  | 0.315 |
| **Neutrophils, x10^9^/L** |  | 2.5(0.0-250.0) |  | 2.2(0.0-231.2) |  | 0.213 |
| **LDH, U/L** |  | 355.6(44.7-7286.0) |  | 346.5(128.0-7286.0) |  | 0.243 |
| **CR after 2 courses** |  |  |  |  |  | 0.612 |
| **Yes** |  | 205(59.6) |  | 40(56.3) |  |  |
| **No** |  | 139(40.4) |  | 31(43.7) |  |  |
| **HCT** |  |  |  |  |  | 0.059 |
| **Yes** |  | 39(11.3) |  | 3(4.1) |  |  |
| **No** |  | 305(88.7) |  | 71(95.9) |  |  |

**NOTE.** Bold font indicates statistical significance.

**Abbreviations:** BSA, body surface area; BMI, Body Mass Index; FAB, French-American-British(classification); R882, Arginine 882; *FLT3*-ITD, *fms-like tyrosine kinase3*-intenal tandem duplication; AraC, cytarabine; ACLA, aclarubicin; G-CSF, granulocyte-colony stimulating factor; THP, pirarubicin; BM, bone marrow; PB, peripheral blood; WBC, white blood cell; RBC, red blood cell; LDH, lactate dehydrogenase; CR, complete remission; HCT, hematopoietic cell transplantation.
